# Design of Bio-Absorbent Systems for the Removal of Hydrocarbons from Industrial Wastewater: Pilot-Plant Scale

**DOI:** 10.3390/toxics9070162

**Published:** 2021-07-07

**Authors:** Gloria Andrea Silva-Castro, Alfonso Rodríguez-Calvo, Tatiana Robledo-Mahón, Elisabet Aranda, Jesús González-López, Concepción Calvo

**Affiliations:** 1 Institute of Water Research, University of Granada, 18071 Granada, Spain; gloria.silva@eez.csic.es (G.A.S.-C.); arcalvo@ugr.es (A.R.-C.); robledo_mahon@af.czu.cz (T.R.-M.); earanda@ugr.es (E.A.); jgl@ugr.es (J.G.-L.); 2 Department of Microbiology, Pharmacy Faculty, Campus de Cartuja s/n, University of Granada, 18071 Granada, Spain

**Keywords:** hydrocarbons, bioremediation, industrial wastewater, pilot scale, microbial diversity, biofilm

## Abstract

The objective of this study was the development and design of a treatment system at a pilot-plant scale for the remediation of hydrocarbons in industrial wastewater. The treatment consists of a combined approach of absorption and biodegradation to obtain treated water with sufficient quality to be reused in fire defense systems (FDSs). The plant consists of four vertical flow columns (bioreactors) made of stainless steel (ATEX Standard) with dimensions of 1.65 × 0.5 m and water volumes of 192.4 L. Each bioreactor includes a holder to contain the absorbent material (Pad Sentec polypropylene). The effectiveness of the treatment system has been studied in wastewater with high and low pollutant loads (concentrations higher than 60,000 mg L^−1^ of total petroleum hydrocarbons (TPH) and lower than 500 mg L^−1^ of TPHs, respectively). The pilot-plant design can function at two different flow rates, Q1 (180 L h^−1^) and Q2 (780 L h^−1^), with or without additional aeration. The results obtained for strongly polluted wastewaters showed that, at low flow rates, additional aeration enhanced hydrocarbon removal, while aeration was unnecessary at high flow rates. For wastewater with a low pollutant load, we selected a flow rate of 780 L h^−1^ without aeration. Different recirculation times were also tested along with the application of a post-treatment lasting 7 days inside the bioreactor without recirculation. The microbial diversity studies showed similar populations of bacteria and fungi in the inlet and outlet wastewater. Likewise, high similarity indices were observed between the adhered and suspended biomass within the bioreactors. The results showed that the setup and optimization of the reactor represent a step forward in the application of bioremediation processes at an industrial/large scale.

## 1. Introduction

Hydrocarbons are a large group of organic compounds ubiquitous in air, soil, and water and can be classified into saturated, unsaturated, and aromatic varieties. The complexity of the hydrocarbon molecule will determine its toxicity, its persistence in the environment, and its degradability. Immunotoxicity, cardiotoxicity, and carcinogenicity are some of the adverse effects caused by hydrocarbons [1]. For example, the polycyclic aromatic hydrocarbons (PAHs) in aquatic ecosystems are harmful to fish, benthic invertebrates, and marine vertebrates [2]. Likewise, the presence of total petroleum hydrocarbons (TPHs) in the soil likely inhibits seed germination and affects plant growth. For instance, it was reported that 1.5% TPH is the critical value for plant growth [3]. Lastly, some hydrocarbons such as PAHs can act as carcinogenic, mutagenic, and immunosuppressant pollutants. Nevertheless, not all petroleum hydrocarbons and their derivatives possess the same toxicity and recalcitrance and are influenced by different chemical properties such as the complexity of the molecule, water solubility, volatility, etc.

The hydrocarbon contamination of water is common. Occasionally, contamination occurs due to accidental events such as shipwrecks, the breakdown of oil pipelines, or ruptures of the storage tanks, which usually cause the spillage of large amounts of pollutants [4,5,6]. However, industrial activities such as the storage, transport, and distribution of hydrocarbon derivatives and the cleaning activities of the relevant facilities are the most common origins of water pollution [7,8]. Water that is chronically polluted by industrial activities does not usually contain high concentrations of hydrocarbons and, consequently, can be successfully decontaminated if effective treatments are applied.

Bioremediation is a cleaning technology that uses microorganisms or compounds produced by those organisms to remove pollutants from the ecosystem, thereby restoring the ecosystem’s quality. Microorganisms can degrade the majority of hydrocarbons, and autochthonous microbial degraders are often naturally augmented in the ecosystem after the contaminant appears. The use of bioremediation for cleaning soil and water contaminated with hydrocarbons is considered to be a successful, environmentally friendly, and cost-effective treatment [9,10].

In water, microorganisms can be as planktonic microbiota or adhered to biotic or abiotic surfaces forming complex microbial communities called biofilms. In these communities, microorganisms have a greater chance to survive, especially in periods of stress, since they are protected against environmental changes within the polysaccharide matrix, have greater accessibility to nutrients, and establish microbial consortia with a wider range of metabolic activities, thereby allowing the microorganisms to degrade a great diversity of pollutants [11].

In addition, a gradient of oxygen, water content, and nutrient availability is established along the biofilm matrix, resulting in the distribution of microbial communities according to their physiological and metabolic characteristics. In the biofilm, the competition established between different members of the microbial community, bacteria, and fungi facilitates the formation of microbial consortia suitable for bioremediation [12]. Bioremediation is facilitated by the fact that the exopolysaccharide (EPS) matrix in which the microorganisms are immobilized also retains the contaminants that will be modified, leading to a dynamic shift in the microbial population and, as a consequence, dynamic degradation [13]. Thus, immobilized microorganisms attached to a carrier forming a biofilm have been successfully used in aquatic ecosystems [14]. Accordingly, immobilization of the biomass represents an effective method for retaining degrading microorganisms, such as hydrocarbon degraders.

The Circular Economy and the Green Deal proposed by the EU for 2050 call for all productive sectors to avoid pollution, remediate contaminated locations, and reuse those locations to achieve a lower consumption of resources. Companies in the fuel industry involved in crude extraction, refining processes, storage, and the transportation of crude and its derivative compounds must take special care in controlling the pollution derived from their activities. These facilities usually produce large volumes of wastewater contaminated with hydrocarbons as a result of their cleaning tasks, the discharge of storage tanks, and small leaks [15,16].

The aim of this research is to treat hydrocarbon industrial wastewater at a prototype scale using a biosorbent system with the capacity to retain a high number of hydrocarbons and create a stable microbial biofilm with strong degradation abilities. Previous studies have demonstrated the efficacy of biofilm bioreactors using Corksorb and Pad Sentec™ hydrophobic sorbents for the decontamination of hydrocarbon-polluted wastewater [17]. In this study, the influence of operational parameters such as flow rate, aeration, and hydraulic retention time was analyzed to determine the most suitable conditions for hydrocarbon removal. To carry out this research, a biofilm bioreactor with Pad Sentec™ as the sorbent material was designed and built at a pilot-plant scale. Gravimetric TPH and hydrocarbon fractions, analyzed by gas chromatography/mass spectrometry (GC/MS), were used to monitor the remediation process. In addition, the relative abundance of bacterial and fungal populations inside the bioreactor, including both planktonic microorganisms and those that adhered to the carrier surface forming the biofilm, was compared with the abundance in the influent wastewater and the effluent-treated water. 

## 2. Materials and Methods

### 2.1. Design and Construction of the Pilot Plant

**Figure 1:**
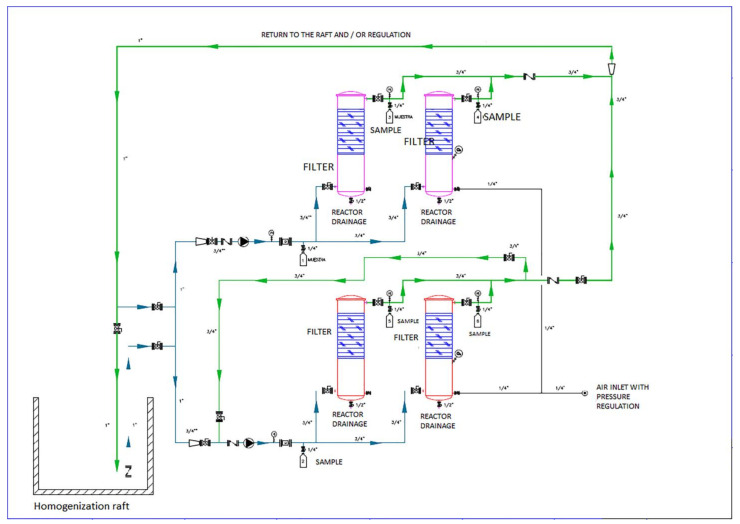
The scheme of the pilot plant used in these studies.

The experiments in this study were carried out in a pilot plant installed in the facility of the Compañía Logística de Hidrocarburos (CLH Company) in Motril (Granada, Spain). Figure 1 shows the scheme of the pilot plant used in these studies. The pilot plant consisted of 4 vertical flow columns of 1.65 × 0.5 m made from stainless steel (ATEX Standard). A basket case was placed inside each bioreactor to hold the sorbent material (a polypropylene melt-blown hydrophobic sorbent supplied by the company SENTEC S.L.). The sorbent material was rolled up as a coil, and the area occupied by the absorbent material was 45% of the total height. The filling material was composed of 99.7% melt-blown polypropylene and 0.3% blue pigment.

Hydrocarbon industrial wastewater (192.4 L) was recirculated through the sorbent material in an upward flow from the bottom of each bioreactor via peristaltic pumps in closed recirculation cycles. Two of the bioreactors, bioreactors 1 (B1) and 2 (B2), worked at a low flow rate, Q1 (180 L h^−1^), and the other two (B3 and B4) worked at a high flow rate, Q2 (780 L h^−1^). Bioreactor 2 (B2) and bioreactor 4 (B4) featured additional aeration (5 mg L^−1^), with one for each flow rate. Aeration of the bioreactors was carried out using a diffuser fed with a pump.

The wastewater was pumped from the API (American Petroleum Industry) storage tank to each bioreactor via peristaltic pumps at a 180 L h^−1^ flow rate for bioreactors B1 and B2 and 780 L h^−1^ for bioreactors B3 and B4. We also installed a series of valves to redirect the flow path, depending on the working conditions, as well as flow meters, pressure devices, and a control panel to control the operating conditions.

Throughout this study, two types of water samples were used to assess the efficacy of the biotreatment system. A set of samples with TPH concentrations between 68,077.67 and 86,530.00 was used in the first phase of this research (high load of hydrocarbons), and another set of water samples with lower hydrocarbon content was tested with TPH concentrations ranging between 70 and 140 mg L^−1^. The four bioreactors worked simultaneously with a volume of water totaling 192.4 L in each and Pad Sentec™ as the sorbent material.

### 2.2. Hydrocarbon Analyses

Total petroleum hydrocarbon (TPHs) from the influent (IWW) and effluent (OWW) wastewater samples were extracted with a 1:1 mixture of hexane:acetone and quantified via gravimetric analyses. The hydrocarbon fractions were determined by GC/MS as previously described [8].

### 2.3. Scanning Electron Microscopy (SEM)

Samples of sorbent material were analyzed by scanning electron microscopy following the methodology previously described by [8]. The analyses were carried out using LEO 1430VP and LEO 1430VP microscopes equipped with an INCA350 EDX system at the Center for Scientific Instrumentation of the University of Granada (Spain).

### 2.4. Microbial Diversity

We studied microbial diversity in the IWW and OWW water samples, as well as in-side the bioreactors (suspended and adhered biomass). All samples were subjected to DNA extraction using a FastDNA SPIN kit for soil (MP Biomedical, Solon, OH, USA) according to the manufacture’s protocol. Before extraction, the samples were pre-concentrated by centrifugation (for the water samples) and sonication and centrifugation (for the sorbent material) under the conditions previously described by [18]. DNA samples were subjected to a massive sequencing procedure using Illumina MiSeq technology, following the protocol described by [19,20]. Primers 515F and 806R were used for the V4 region of the 16S rRNA gene [21,22], while ITS1 and ITS2 were used for the fungal microbiota [23]. Sequencing analyses were performed using the microbial ecology software QIIME2, version 2017.62. Initially, the sequences were analyzed and trimmed to eliminate low-quality reads. Then, Alpha–Beta diversity analyses and taxonomic analyses were performed.

### 2.5. Processing of Sequencing Data

For the taxonomic analysis, Greengenes 13_8 with 99% operational taxonomic units (OTU) was applied for the bacterial microbiota. For the fungal microbiota, UNITE (fungal ITS) was applied as a classifier, as previously described [18]. The β-diversity was calculated using Bray–Curtis and Uni-Frac [24] distance algorithms for bacterial and fungal communities, respectively, and presentation was performed via non-metric multidimensional scaling (NMDS) using the Emperor software [25] and PRIMER 6.

### 2.6. Statistical Analyses

The mean and standard deviation of the parameters used were calculated from the values of the triplicate samples. Non-metric multidimensional scale analysis (MDS) was used to determine the distances between the bioreactor experiments. The correlation matrix was determined using the MDS ALSCAL algorithm, with the Euclidean model was applied to calculate the optimal distances between the studied operating parameters. All statistical analyses were carried out using the IBM SPSS (Statistical Package for the Social Sciences) software.

## 3. Results and Discussion

### 3.1. Bioreactor Experiments

The pilot plant was dimensioned based on the previous results obtained at a lab-scale pilot plant [17]. The experiments were carried out to determine the most suitable operational parameters to achieve maximum efficacy. In all experiments, the flow inlet was located at the bottom of the column. In this way, the inlet wastewater could rise and flow through the Pad Sentec™ sorbent, enabling the retention of hydrocarbons and the adherence of autochthonous microorganisms from the wastewater to form a stable biofilm [8].

**Figure 2:**
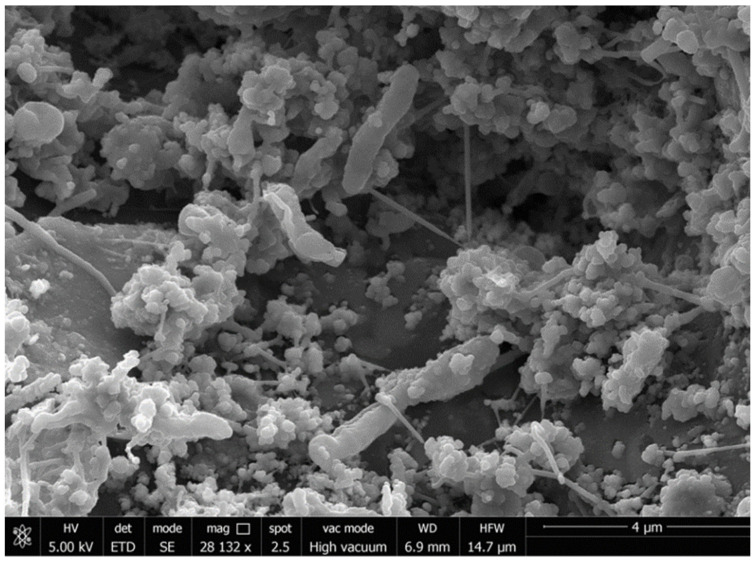

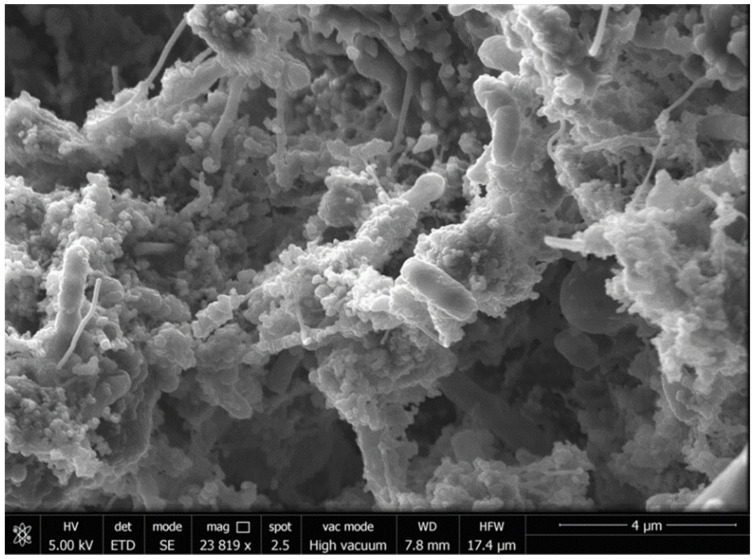
SEM images. General view of bacteria adhering to the surface of the sorbent material into the bioreactor showing the biofilm formed into the bioreactor.

Figure 2 shows the biofilm formed by microorganisms adhering to the surface of Pad Sentec™ sorbent inside the bioreactor. Previous studies carried out at the lab scale [17] showed that the hydrocarbon removal in these systems is a consequence of the joint action of the adsorbent capacity of the filler material (Pad Sentec™ sorbent) and the biomass (biofilm) that develops on the material. Previous tests carried out in the laboratory showed that to start treatment plant operations, the system needs sufficient conditioning time for the microorganisms to form a stable biofilm on the surface of the absorbent material. Therefore, to create a stable biofilm in the bioreactors, wastewaters containing high or low hydrocarbon concentrations were recirculated for a period of 1 h applying a flow rate of 780 L h^−1^. After this recirculation period, the bioreactor was maintained at rest for 4 weeks. After this period, the formation of a stable biofilm was verified by SEM analyses (Figure 2). Based on these analyses, it was determined that the system had reached a level of microbial stability sufficient for the biotreatment. Later, the most suitable operating conditions were established for the wastewater treatment. These properties ensure that a stable biofilm is consolidated and that the bioreactor will work properly. The capacity of autochthonous microorganisms from hydrocarbon industrial wastewater to adhere to the surface of the Pad Sentec^TM^ sorbent was previously demonstrated in a microcosm and at a lab-scale pilot plant [8,17]. In addition, the hydrocarbon absorbed on sorbent during by the treatment was shown to be susceptible to biodegradation through the autochthonous microorganisms adhering to the sorbent filling material [17].

Although the wastewater that is usually treated in these facilities has concentrations of TPH lower than 1000 mg L^−1^, for the present study, wastewater samples with high concentrations of hydrocarbons—86,530.00 ± 38,565.84 mg L^−1^ for B1 and B2 and 68,077.67 ± 24,599.93 mg L^−1^ for B3 and B4—were selected to demonstrate the effectiveness of the system and the resilience of the process against the accidental appearance of a high pollutant load. The results obtained are detailed in Table 1. The four bioreactors showed high efficiency in TPH removal for the majority of hydrocarbon fractions analyzed. After 24 h of treatment, the percentages of removal ranged between 90 and 100 in bioreactors working at low and high flow rates, with or without aeration.

When the treatment included eight recirculation cycles for the total bioreactor volume, certain differences were observed in the hydrocarbon removal capacity of the system. Particularly, for B1 (working without aeration and at a flow rate of 180 L h^−1^), the removal percentage of c10–c20 and branched/cyclic alkanes were 19.05% and 25.11%, respectively, and the percentage of TPH elimination was 70.0 (Table 1). However, in B2 (working at the same flow rate but with aeration), the efficacy of biotreatment was noticeably enhanced for all the hydrocarbon fractions reaching between 83% and 99% removal, suggesting that when the bioreactor works at a low flow rate, air supplementation could be necessary to enhance hydrocarbon removal.

**Figure 3:**
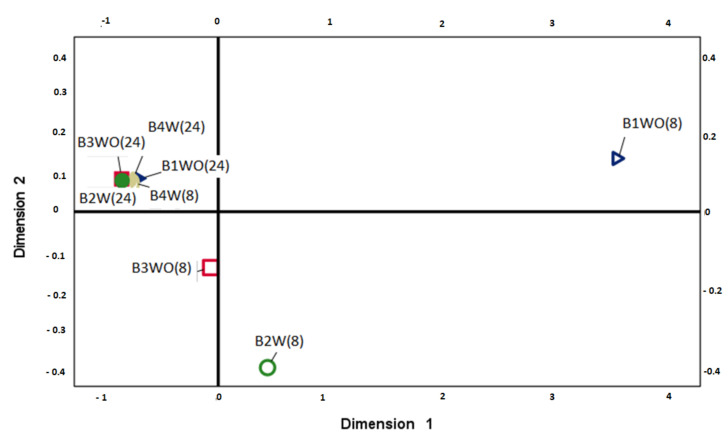
Optimal two-dimensional matrix generated by MSD correlation. B1(8c): (180 L h^−1^; non-aerated; eight recirculation cycles). B1(24 h): (180 L h^−1^; non-aerated; 24 h of treatment). B2(8c): (180 L h^−1^; aerated; eight recirculation cycles). B2(24 h): (180 L h^−1^; aerated; 24 h of treatment). B3(8c): (780 L h^−1^; non-aerated; eight recirculation cycles). B3(24 h): (780 L h^−1^; non-aerated; 24 h of treatment). B4(8c): (780 L h^−1^; aerated; eight recirculation cycles). B4(24 h): (780 L h^−1^; aerated; 24 h of treatment).

B3 and B4, which worked at a high flow rate (780 L h^−1^), were more effective in the hydrocarbon wastewater treatment than those working at a low flow rate (180 L h^−1^). At high flow rate, aeration did not provide a noticeable advantage compared to the non-aerated bioreactor, with both reaching values close to 90% or the upper limit of removal (Table 1). Figure 3 shows the distribution of the experimental setups’ performance in removing different fractions of TPH of wastewater with a high pollutant load was carried out. The treatments that used 24 h of operation, as well as B4 (8c), did not show differences between the concentrations of the hydrocarbon fractions analyzed, being located in the same left quadrant. On the contrary, B1 (8c) and B2 (8c) showed a distance with the other treatments due to their low performance in the operating conditions with eight recirculation cycles. On the other hand, B3 with eight cycles of recirculation and 780 L h^−1^ flow rate without forced aeration achieved high percentages of hydrocarbon reduction, suggesting that these conditions can reduce treatment times and obtain optimal decontamination levels.

The Pad Sentec^TM^ sorbent utilized as the support material inside the bioreactors worked as a highly efficient submerged filter against high concentrations of hydrocarbons. In addition, it showed no detachment from the biofilm and there was no deterioration of the absorbent material, as reported previously [8]. The effectiveness of submerged filter technology in industrial wastewater treatment was highlighted by several authors [26,27,28].

In the second group of experiments, the bio-adsorption system was studied using wastewater containing low concentrations of hydrocarbons, since such concentrations are normally found in wastewater. On this occasion, and considering the results obtained in previous studies mention before, it was considered more appropriate to work with a flow rate of 780 L h^−1^ without supplementary aeration. The experiment was carried out under both recirculation and non-recirculation conditions.

Inlet wastewater was collected from the API storage tank at a 1.30 m depth. Although the concentration of hydrocarbon fractions varied among the different wastewater samples studied, the inlet wastewater samples were characterized by a low content of hydrocarbons with values of TPH ranging between 75 and 135 mg L^−1^ and concentrations of C10–C40 alkanes between 9.6 and 51.8 mg L^−1^. Similar averages were observed in other hydrocarbon fractions as they were branched and cyclic alkanes between 2.5 and 20.1 mg L^−1^ and pregnanes with values ranging between 6.5 and 20.3 mg L^−1^. As previously mentioned, to restart the bioreactor for this second stage of the study, wastewater was recirculated through the bioreactor successively for one week. In this way, stabilization of the system was achieved.

Table 2 summarizes the results obtained in the four trials carried out to evaluate the efficacy of the bioreactor in terms of hydrocarbon removal when the wastewater was recirculated through the biosorbent over three and four cycles of recirculation. The quantity of hydrocarbons removed was similar under both scenarios, although for some hydrocarbon fractions, such as the c20–c40 alkanes and alkene compounds, the concentrations detected in the four-cycle outlet water samples were slightly lower than those in the three-cycle outlet-treated water samples.

We also analyzed the efficiency of an additional treatment that involved leaving the wastewater inside the bioreactor for 7 days without recirculation. Briefly, the wastewater samples were recirculated four times through the sorbent material and then left to rest for 7 days to promote the bioremediation process. Analyses of the outlet water samples showed differences between the four experiments. In trial 1, the post-treatment produced a noticeable decrease in hydrocarbons, specifically in the c10–c20, c20–c40, and pregnanes compared to the amounts detected in the outlet water samples after four cycles of recirculation. In trial 4, post-treatment also enhanced the biodegradation of all hydrocarbons. In contrast, during trials 2 and 3, the removal efficacies were similar to those detected after four cycles of recirculation. The decrease in hydrocarbons during this period of post-treatment under resting conditions can be attributed to the process of microbial biodegradation.

**Figure 4:**
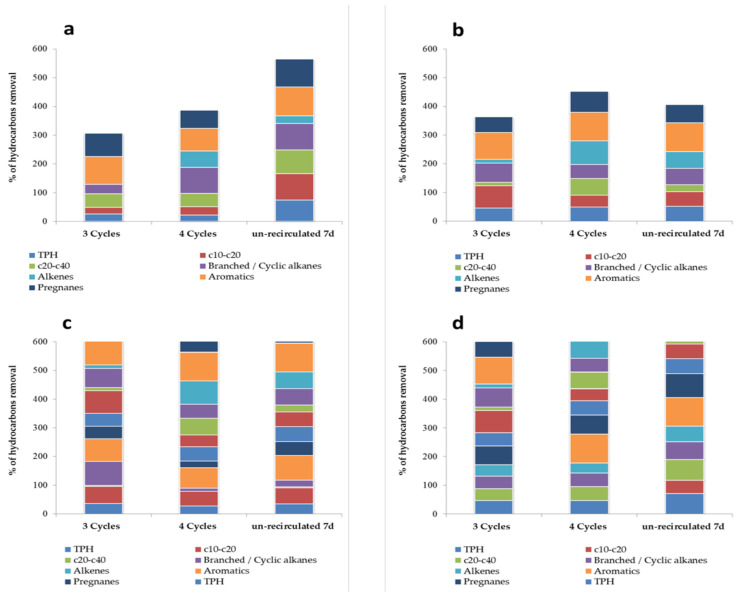
Sum of the percentages of hydrocarbons removal after three and four cycles of recirculation and after four cycles of recirculation plus 7 days of post-treatment in rest conditions. (**a**–**d**) Trials 1–4.

Figure 4 shows the sum of the percentages of hydrocarbon fractions and TPH removal in each of the experimental conditions tested (trials 1–4). This figure also shows the weigh that degraded in each fraction compared to the total. Experiments 1 and 4 clearly show an increase in degradation after 7 days of post-treatment. These differences were not observed in trials 2 and 3. Likely, the behaviors of fractions c10–20 and c20–40 were different for tests 1 and 4, in which the degradation of c20–40 predominated at the beginning of treatment (three cycles). Conversely, in trials 2 and 3, the c10–c20 fraction decreased more sharply in the effluent after three cycles of recirculation.

**Figure 5:**
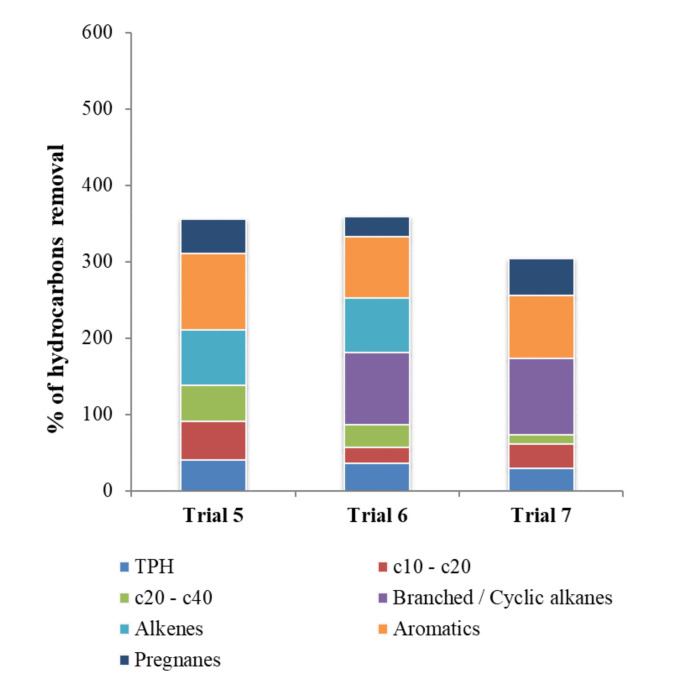
Sum of the percentages of hydrocarbons removal after 24 h of recirculation in trials 5, 6, and 7.

The following experiments were carried out to analyze the efficiency of the bioreactor working for 24 h with continuous recirculation. The results obtained are summarized in Table 3. The quantity of hydrocarbons decreased after the treatment in all the experiments. However, a longer duration of treatment did not produce a related increase in the biodegradation of the hydrocarbons. As shown in Figure 5, which illustrates the accumulated percentage of degradation among all the hydrocarbon fractions, including the TPHs, the achieved degradation rates were generally no higher than the values reached after three and four recirculation cycles.

In summary, the results obtained in the four trials carried when the wastewater was recirculated through the biosorbent over three and four cycles of recirculation showed that the concentration of hydrocarbons was similar under both scenarios. We also analyzed the efficiency of an additional treatment that involved leaving the wastewater inside the bioreactor for 7 days without recirculation. The decrease in hydrocarbons during this period of post-treatment under resting conditions can be attributed to the process of microbial biodegradation

### 3.2. Microbial Diversity

As mentioned previously, bioremediation was largely responsible for the removal of hydrocarbons in the studied wastewater treatment system. In this system of treatment, the microbial consortium that degraded the pollutants came from the wastewater contaminated by the hydrocarbons that were treated. The autochthonous microorganisms were enriched and selected in response to the environmental conditions of the system to build the microbial consortia. The composition of the microbial population is one of the factors that shapes the dynamics of the biodegradation process. The microbial diversity of the IWW and OWW samples was compared with the microbial diversity inside the bioreactor. Within the bioreactor, we analyzed the microorganisms adhering to the sorbent surface forming the biofilm (Pad) and the planktonic microorganisms suspended in the water that remained inside the bioreactor (InW). These samples were taken in B3 (780 L h^−1^; non-aerated; 8 recirculation cycles) after the four trials.

**Figure 6:**
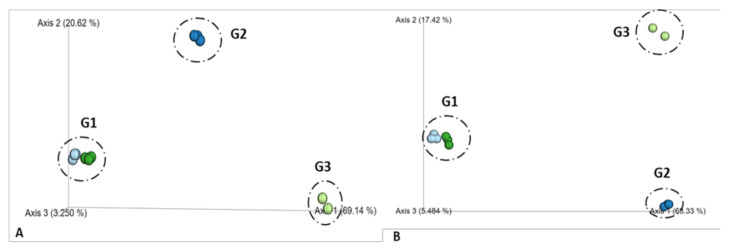
Beta diversity analysis in ordination plot (PCoA) of the Bray–Curtis dissimilarity matrix associated with influent and effluent water (G1), water inside the bioreactor (G2), and in the Pad Sentec (G3). (**A**): bacterial communities; (**B**): fungi communities.

The beta diversity approach was used to determine the differences in microbial composition based on the abundance of the dominant species in each of the analyzed samples. The Bray–Curtis parameter of similarity was used to obtain the distribution of the samples and the principal coordinate plot (PCoA) to determine whether there was a clear separation between them. The ordination of the Bray–Curtis dissimilarity matrix of bacterial communities described 89.7% of the total variability in all samples (69.14% and 20.62% for the first and second axes, respectively). For fungi communities, the two main axes indicate 68.33% for the first axis and 17.42% for the second axis. In both cases, the samples are plotted into three groups separated by the origin of the samples: influent and effluent water (G1), water inside the bioreactor (G2), and biofilm in the Pad Sentec (G3) (Figure 6).

To assess the differences found in the bacterial and fungal communities based on the beta diversity of the studied samples and determine the different communities involved in the wastewater treatment at the species level, a taxonomic analysis was carried out.

**Figure 7:**
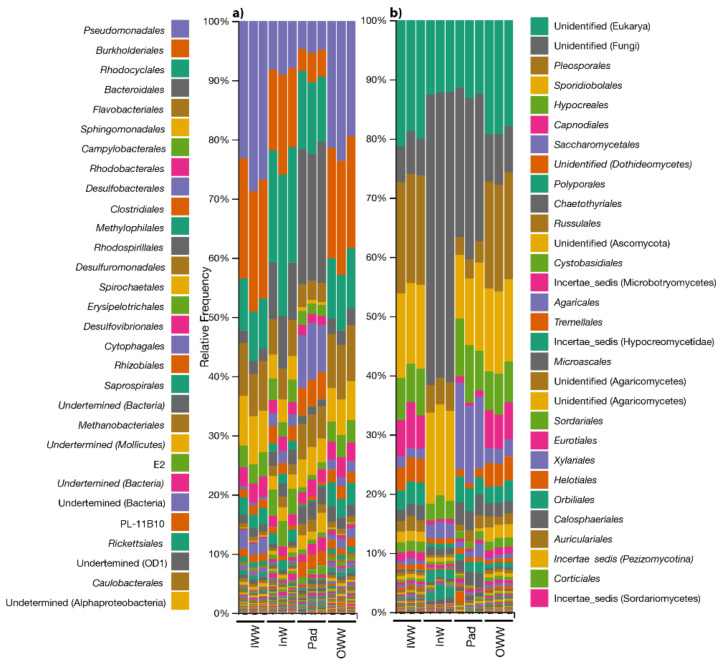
Relative abundance in percentage (%) of (**a**) bacterial and (**b**) fungal communities at the order level in the inlet wastewater samples (IWW), outlet wastewater samples (OWW), wastewater samples inside the bioreactor (InW), and adhered on the surface of Pad Sentec carrier (Pad). Only percentages of abundance ≥1% are represented. Samples were analyses in triplicates.

Figure 7a shows the relative abundance of bacterial orders in the IWW and OWW, InW, and Pad samples. Sequencing of the bacterial community showed that *Pseudomonadales*, *Burkholderiales*, *Rhodocyclales*, and *Bacteroidales* were the predominant orders in all the samples. Member of the *Pseudomonas* genus have been demonstrated to produce biosurfactants that can facilitate the pseudo-solubilization of hydrocarbons [29]. The action of the biosurfactant may improve the bioavailability of hydrocarbon substrates to be attacked by microbial activity [30]. Therefore, hydrocarbon degradation involves a cooperative microbial network where the metabolites produced can be used by other microorganisms, increasing the biological activity of the system [31]. In some cases, these microorganisms are favorable in forming a biofilm. Previous studies have reported good results in achieving hydrocarbon degradation by using biofilm in sorbent materials [8,17]. The *Pseudomodales* and *Burkholderiales* orders were predominant in the IWW and OWW samples, with percentages of relative abundance between 26% and 21.5%. This group de-creased to 8% and 5% in the InW and Pad samples, respectively. *Burkholderiales* likely decreased from 20% in the IWW and OWW samples to 4.4% in the Pad samples. In contrast, the *Rhodocyclales* (close to 21%) and *Bacteroidales* orders (close to 22%) were predominant in the InW and Pad samples.

The community forming a biofilm was analyzed in the Pad sample. Comparing the bacterial communities in the Pad and InW samples, *Burkholderiales* and *Rhodocyclales* presented a higher percentage of relative abundance in the InW samples (around 10% and 20%, respectively) than in the Pad samples (less than 5% and 10%, respectively). Conversely, *Bacteroidales* were more abundant in the Pad samples (around 20%) than in the InW samples (around 10%). The percentages of *Desulfobacterales* were also higher in the Pad samples (around 8%) than in the InW samples. This trend was also observed in the fungal community, where the greatest differences in composition were found between InW and Pad. These results may indicate that the biofilm that adhered to the Pad favored *Bacteroidales*, whereas *Burkholderiales* and *Rhodocyclales* were favored in the suspended water, as bacteria of these orders could be part of the external portion of the biofilm in contact with the suspended water. Previous studies [32] reported changes in the bacterial community structure when biofilm was grown on the oil–water interface, due to a depletion of oxygen in the internal portion of the generated biofilm and the environment’s association with anaerobic microorganisms, as was also the case for *Bacteroidales* [33]. 

There were no significant differences between the bacterial communities in terms of the incoming water (IWW) and the outcoming water (OWW). However, *Pseudomonadales* was reduced within the bioreactor, with 20% present in the IWW, which decreased to below 10% in the InW sample within the bioreactor. The bacterial composition was similar between IWW and OWW in terms of relative abundance, but the conditions inside the bioreactor favored the proliferation of *Bacteroiodales* on the sorbent in the Pad sample and *Rhodocyclales* in the InW sample, to the detriment of *Pseudomonades.*

High-throughput sequencing of fungal ITS2 rDNA from the 12 samples resulted in 992,700 reads, among which 903,914 were of high quality. These non-chimeric reads were pooled and clustered into 3178 fungal features. The identity threshold was 97%. *Ascomycota* represented the predominant phylum, with a 40% variation of abundance in IWW, 20% in InW, 35% in Pad, and 47% in the OWW. 

*Basidiomycota* was the second most abundant division, with percentages of 26%, 18%, 26%, and 26% for IWW, InW, Pad, and OWW, respectively. Notably, around 26% and 36% of the features were undefined in all the samples, particularly in the InW sample in which the number of undefined features reached 60%. The other identified divisions belonged to *Chytridiomycota*, *Rozellomycota*, and *Zygomycota*, with percentages of less than 0.5% in terms of relative abundance.

These data agree with other studies showing a predominance of Ascomycota fungi in environments polluted with hydrocarbons [34]; these fungi could play an important role in hydrocarbon removal [35,36]. 

At the order level, the relative abundance of *Pleosporales* was greater in IWW and OWW (18%) than in InW and Pad (3%). *Sporidiobolales* remained constant in all the samples (11–14%). 

*Capnodiales* was more abundant in the IWW and OWW (around 7%) samples than in the interior and Pad samples (1%). In general, the populations in the IWW and OWW samples were more similar to each other than to the populations in the InW and Pad samples. *Saccharomycetales* was predominant in the Pad samples, with a relative abundance of 12%. At the genus level, *Rhodotorula* (*Sporidiobolales*), *Didymella* (*Pleosporales*), and *Candida* (*Sacharomycetalles*) were the most abundant genera in all the systems. These are unicellular fungi belonging to *Basidiomycota* and *Ascomycota* that have been previously shown to form biofilms, which could explain the abundance of these fungi in the Pad samples [37,38]. Despite most studies on fungal communities focusing on hydrocarbon-polluted soils, similar communities have also been found in wastewater. Such communities were previously described as degraders of hydrocarbons, as well as aliphatic and aromatic hydrocarbons.

## 4. Conclusions

The results of this study show that the system under consideration represents an effective technology for removing hydrocarbons from industrial wastewater; this technology is also easy to handle and implement. The results indicate that the effectiveness of this system is based on the adsorbent capacity of polypropylene fibers and the formation of a specialized microbial consortium by the bacterial and fungal community that we characterized. The results represent an important advancement in the application of this technology to obtain high-quality treated water for reuse under real conditions.

## Figures and Tables

**Table 1:** Efficacy in TPH removal of bioreactors 1, 2, 3, and 4 after eight recirculation cycles and after 24 h of treatment in continuous recirculation. B1 and B2 were working at a flow rate of 180 L h^−1^, and B3 and B4 at flow rate of 780 L h^−1^.

	Hydrocarbon Concentration in mg L^−1^			% of Removal	
	IWW	OWW 8 Cycles	OWW 24 h	8 Cycles	24 h
Bioreactor 1					
TPH	86,530 ± 8565	25,933 ± 4397	1813 ± 95	70.0	97.9
c10–c20	10,619 ± 6135	8596 ± 453	673 ± 127	19.1	93.7
c20–c40	4399 ± 768	1383 ± 1669	241 ± 78	68.6	94.5
Branched/Cyclic alkanes	800 ± 504	599 ± 120	25.1 ± 16.5	25.1	96.9
Alkenes	ND ^1^	ND	ND	ND	ND
Aromatics	468.96 ± 185.42	19.5 ± 4.8	ND	95.8	100
Pregnanes	6484 ± 2491	438.7 ± 146.7	29.2 ± 11.5	93.2	99.6
Bioreactor 2					
TPH	86,530 ± 38,565	9196 ± 2338	950.0 ± 85.4	83.4	98.9
c10–c20	10,619 ± 6135	7.15 ± 1.27	374 ± 125	99.9	96.5
c20–c40	4399 ± 768	156.1 ± 24.4	140.2 ± 26.1	96.5	96.8
Branched/Cyclic alkanes	800 ± 504	3.6 ± 2.9	6.7 ± 8.2	99.6	99.2
Alkenes	ND	ND	ND	ND	ND
Aromatics	468 ± 185	1.22 ± 0.21	ND	99.7	100
Pregnanes	6484 ± 2491	507 ± 108	7.2 ± 5.7	92.2	99.9
Bioreactor 3					
TPH	68,077 ± 24,599	5227 ± 1072	920 ± 173	92.32	98.7
c10–c20	9073 ± 1317	754 ± 394	395.7 ± 21.8	91.69	95.6
c20–c40	1301 ± 404	223 ± 87	102.2 ± 11.3	82.88	92.2
Branched/Cyclic alkanes	792 ± 71	65.2 ± 12.4	7.0 ± 1.1	91.76	99.1
Alkenes	1075 ± 161	ND	ND	100	100
Aromatics	158 ± 26	9.4 ± 2.5	4.1 ± 0.2	94.03	97.4
Pregnanes	1675 ± 151	370 ± 167	14.7 ± 1.9	77.92	99.1
Bioreactor 4					
TPH	68,077 ± 24,599	1607 ± 307	1293 ± 349	97.64	98.1
c10–c20	9073 ± 1317	545 ± 126	570 ± 5	93.99	93.7
c20–c40	1301 ± 404	140.4 ± 34.6	170 ± 19	89.21	86.9
Branched/Cyclic alkanes	792 ± 70	11.77 ± 4.37	4.19 ± 5.92	98.52	99.5
Alkenes	1075 ± 161	ND	ND	100	100
Aromatics	158 ± 26	4.6 ± 0.1	4.7 ± 1.0	97.11	97.0
Pregnanes	1675.34 ± 151.30	72.9 ± 10.5	46 ± 3	95.65	97.3

^1^ ND, not detected. ± from the triplicate of the samples.

**Table 2:** Hydrocarbons concentration in mg L^−1^ in the IWW samples and in the OWW samples after three and four cycles of recirculation and after 7 days of post-treatment in rest conditions. Trials 1, 2, 3, and 4.

	Hydrocarbon Concentration in mg L^−1^			
	IWW	OWW 3 Cycles	OWW 4 Cycles	OWW Unrecirculated 7 d
Trial 1				
TPH	124.0 ± 28.3	93.0 ± 18.6	97.3 ± 24.1	33.0 ± 4.2
c10–c20	24.5 ± 3.7	19.0 ± 4.7	17.5 ± 0.4	2.0 ± 0.3
c20–c40	27.3 ± 4.1	14.4 ± 2.9	14.5 ± 8.3	4.7 ± 0.01
Branched/Cyclic alkanes	6.6 ± 0.4	4.4 ± 0.9	0.6 ± 0.3	0.5 ± 2.8
Alkenes	13.4 ± 0.5	13.8 ± 2.7	5.8 ± 2.1	9.8 ± 3.5
Aromatics	2.9 ± 0.4	0.10 ± 0.02	0.6 ± 0.3	ND
Pregnanes	20.3 ± 3.0	3.8 ± 0.7	7.5 ± 1.4	0.6 ± 0.2
Trial 2				
TPH	135.0 ± 2.8	73.3 ± 11.5	68.3 ± 10.4	65.0 ± 7.0
c10–c20	11.3 ± 0.3	2.5 ± 1.2	6.6 ± 3.6	5.6 ± 1.3
c20–c40	14.0 ± 3.1	12.4 ± 1.0	5.9 ± 0.6	10.6 ± 2.3
Branched/Cyclic alkanes	20.1 ± 3.1	6.6 ± 2.7	10.4 ± 0.7	8.5 ± 1.1
Alkenes	29.3 ± 15.2	25.5 ± 10.0	5.2 ± 1.7	12.2 ± 2.8
Aromatics	1.7 ± 0.3	0.1 ± 0.05	ND	ND
Pregnanes	6.5 ± 2.8	3.0 ± 1.6	1.8 ± 0.4	2.4 ± 3.8
Trial 3				
TPH	89.5 ± 14.1	57.7 ± 10.4	65.0 ± 10.0	58.8 ± 4.7
c10–c20	12.5 ± 3.9	5.0 ± 0.4	6.2 ± 2.0	5.4 ± 1.0
c20–c40	6.3 ± 1.2	6.1 ± 0.7	6.8 ± 3.0	6.1 ± 1.0
Branched/Cyclic alkanes	2.5 ± 0.7	0.4 ± 0.2	2.3 ± 1.5	2.0 ± 1.0
Alkenes	3.3 ± 1.3	5.1 ± 0.5	4.1 ± 0.5	10.5 ± 3.0
Aromatics	1.4 ± 0.01	0.3 ± 0.2	0.4 ± 0.1	0.2 ± 0.1
Pregnanes	19.6 ± 0.7	11.2 ± 1.1	15.0 ± 2.2	10.0 ± 2.2
Trial 4				
TPH	75.0 ± 7.1	40.0 ± 10.0	40.0 ± 5.0	21.67 ± 2.9
c10–c20	2.8 ± 0.4	3.8 ± 1.4	4.1 ± 0.3	1.5 ± 0.1
c20–c40	6.8 ± 0.2	4.0 ± 0.1	3.5 ± 0.1	1.9 ± 0.1
Branched/Cyclic alkanes	7.6 ± 0.3	4.3 ± 0.1	4.0 ± 0.4	2.9 ± 0.2
Alkenes	13.4 ± 0.1	8.1 ± 0.1	8.5 ± 0.9	6.1 ± 0.1
Aromatics	0.1 ± 0.0	0.1 ± 0.0	0.0 ± 0.0	0.0 ± 0.0
Pregnanes	17.2 ± 6.3	5.8 ± 3.5	5.6 ± 0.9	2.8 ± 1.5

ND, not detected. ± from the triplicate of the samples.

**Table 3:** Hydrocarbons concentration in mg L^−1^ in the inlet wastewater samples and in the outlet water samples after 24 h of recirculation. Trials 5, 6, and 7.

	Trial 5		Trial 6		Trial 7	
	Influent	Effluent	Influent	Effluent	Influent	Effluent
TPH	81.7 ± 7.6	48.3 ± 2.9	51.7 ± 2.9	33.3 ± 7.6	102 ± 18	71.7 ± 5.8
c10–c20	3.9 ± 0.7	1.9 ± 0.2	3.8 ± 0.6	3.0 ± 0.7	7.8 ± 1.2	5.3 ± 0.7
c20–c40	5.4 ± 1.4	2.8 ± 0.4	3.4 + 0.9	2.4 ± 0.7	5.8 ± 1.4	5.1 ± 0.7
Branched/Cyclic alkanes	ND	ND	2.1 ± 0.5	0.1 ± 0.0	0.9 ± 0.3	0.0 ± 0.0
Alkenes	1.1 ± 0.4	0.30 ± 0.05	1.4 ± 0.2	0.40 ± 0.05	0.8 ± 0.3	1.6 ± 0.5
Aromatics	0.4 ± 0.1	ND	0.5 ± 0.2	0.10 ± 0.05	3.5 ± 0.1	0.6 ± 0.2
Pregnanes	14.4 ± 1.8	8.1 ± 0.7	8.4 ± 0.1	6.2 ± 1.0	27.6 ± 6.9	14.3 ± 4.1

ND, not detected. ± from the triplicate of the samples.

## References

[1] Rengarajan T., Rajendran P., Nandakumar N., Lokeshkumar B., Rajendran P., Nishigaki I. (2015). Exposure to Polycyclic Aromatic Hydrocarbons with Special Focus on Cancer. Asian Pac. J. Trop. Biomed..

[2] Mangwani N., Kumari S., Das S. (2017). Marine Bacterial Biofilms in Bioremediation of Polycyclic Aromatic Hydrocarbons (PAHs) Under Terrestrial Condition in a Soil Microcosm. Pedosphere.

[3] Tang J., Wang M., Wang F., Sun Q., Zhou Q. (2011). Eco-Toxicity of Petroleum Hydrocarbon Contaminated Soil. J. Environ. Sci..

[4] Bacosa H.P., Thyng K.M., Plunkett S., Erdner D.L., Liu Z. (2016). The Tarballs on Texas Beaches Following the 2014 Texas City “Y” Spill: Modeling, Chemical, and Microbiological Studies. Mar. Pollut. Bull..

[5] Uad I., Silva-Castro G.A., Pozo C., González-López J., Calvo C. (2010). Biodegradative Potential and Characterization of Bioemulsifiers of Marine Bacteria Isolated from Samples of Seawater, Sediment and Fuel Extracted at 4000 m of Depth (Prestige Wreck). Int. Biodeterior. Biodegrad..

[6] Yin F., John G.F., Hayworth J.S., Clement T.P. (2015). Long-Term Monitoring Data to Describe the Fate of Polycyclic Aromatic Hydrocarbons in Deepwater Horizon Oil Submerged off Alabama’s Beaches. Sci. Total. Environ..

[7] Gargouri B., Karray F., Mhiri N., Aloui F., Sayadi S. (2011). Application of a Continuously Stirred Tank Bioreactor (CSTR) for Bioremediation of Hydrocarbon-Rich Industrial Wastewater Effluents. J. Hazard. Mater..

[8] Rodríguez-Calvo A., Silva-Castro G.A., Robledo-Mahón T., González-López J., Calvo C. (2018). Capacity of Hydrophobic Carriers to Form Biofilm for Removing Hydrocarbons from Polluted Industrial Wastewater: Assay in Microcosms. Water Air Soil Pollut..

[9] Dellagnezze B.M., de Sousa G.V., Martins L.L., Domingos D.F., Limache E.E.G., de Vasconcellos S.P., da Cruz G.F., de Oliveira V.M. (2014). Bioremediation Potential of Microorganisms Derived from Petroleum Reservoirs. Mar. Pollut. Bull..

[10] Silva-Castro G.A., Rodriguez-Calvo A., Laguna J., González-López J., Calvo C. (2016). Autochthonous Microbial Responses and Hydrocarbons Degradation in Polluted Soil during Biostimulating Treatments under Different Soil Moisture. Assay in Pilot Plant. Int. Biodeterior. Biodegrad..

[11] Gieg L.M., Fowler S.J., Berdugo-Clavijo C. (2014). Syntrophic Biodegradation of Hydrocarbon Contaminants. Curr. Opin. Biotechnol..

[12] Mitra A., Mukhopadhyay S., Mitra A., Mukhopadhyay S. (2016). Biofilm Mediated Decontamination of Pollutants from the Environment. AIMS Bioeng..

[13] Sutherland I.W. (2001). The Biofilm Matrix—An Immobilized but Dynamic Microbial Environment. Trends Microbiol..

[14] Leyva-Díaz J.C., González-Martínez A., González-López J., Muñío M.M., Poyatos J.M. (2015). Kinetic Modeling and Microbiological Study of Two-Step Nitrification in a Membrane Bioreactor and Hybrid Moving Bed Biofilm Reactor–Membrane Bioreactor for Wastewater Treatment. Chem. Eng. J..

[15] El-Borai A.M., Eltayeb K.M., Mostafa A.R., El-Assar S.A. (2016). Biodegradation of Industrial Oil-Polluted Wastewater in Egypt by Bacterial Consortium Immobilized in Different Types of Carriers. Pol. J. Environ. Stud..

[16] Jou C.-J.G., Huang G.-C. (2003). A Pilot Study for Oil Refinery Wastewater Treatment Using a Fixed-Film Bioreactor. Adv. Environ. Res..

[17] Rodríguez-Calvo A., Silva-Castro G.A., Olicón-Hernández D.R., González-López J., Calvo C. (2020). Biodegradation and Absorption Technology for Hydrocarbon-Polluted Water Treatment. Appl. Sci..

[18] Gómez-Silván C., Andersen G.L., Calvo C., Aranda E., Robledo-Mahón (2020). Assessment of Bacterial and Fungal Communities in a Full-Scale Thermophilic Sewage Sludge Composting Pile under a Semipermeable Cover. Bioresour. Technol..

[19] Caporaso J.G., Lauber C.L., Walters W.A., Berg-Lyons D., Lozupone C.A., Turnbaugh P.J., Fierer N., Knight R. (2011). Global Patterns of 16S RRNA Diversity at a Depth of Millions of Sequences per Sample. Proc. Natl. Acad. Sci. USA.

[20] Caporaso J.G., Lauber C.L., Walters W.A., Berg-Lyons D., Huntley J., Fierer N., Owens S.M., Betley J., Fraser L., Bauer M. (2012). Ultra-High-Throughput Microbial Community Analysis on the Illumina HiSeq and MiSeq Platforms. ISME J..

[21] Apprill A., McNally S., Parsons R., Weber L. (2015). Minor Revision to V4 Region SSU RRNA 806R Gene Primer Greatly Increases Detection of SAR11 Bacterioplankton. Aquat. Microb. Ecol..

[22] Parada A.E., Needham D.M., Fuhrman J.A. (2016). Every Base Matters: Assessing Small Subunit RRNA Primers for Marine Microbiomes with Mock Communities, Time Series and Global Field Samples. Environ. Microbiol..

[23] White T.J., Bruns T., Lee S., Taylor J., Innis M.A., Gelfand D.H., Sninsky J.J., White T.J. (1990). 38—Amplification and direct sequencing of fungal ribosomal rna genes for phylogenetics. PCR Protocols.

[24] Lozupone C.A., Knight R. Session: OOS 31—From Microbial to Conservation Biology: Exploring Phylogenetic Beta Diversity as a Theoretical Tool Uniting Disciplines. https://eco.confex.com/eco/2010/techprogram/S5605.HTM.

[25] Vázquez-Baeza Y., Pirrung M., Gonzalez A., Knight R. (2013). EMPeror: A Tool for Visualizing High-Throughput Microbial Community Data. GigaScience.

[26] Bayat A., Aghamiri S.F., Moheb A., Vakili-Nezhaad G.R. (2005). Oil Spill Cleanup from Sea Water by Sorbent Materials. Chem. Eng. Technol..

[27] Gómez M.A., González-López J., Hontoria-García E. (2000). Influence of Carbon Source on Nitrate Removal of Contaminated Groundwater in a Denitrifying Submerged Filter. J. Hazard. Mater..

[28] Bazargan A., Hui C.W., Mckay G. (2014). Marine Residual Fuel Sorption and Desorption Kinetics by Alkali Treated Rice Husks. Cellulose.

[29] Ebadi A., Khoshkholgh Sima N.A., Olamaee M., Hashemi M., Ghorbani Nasrabadi R. (2017). Effective Bioremediation of a Petroleum-Polluted Saline Soil by a Surfactant-Producing Pseudomonas Aeruginosa Consortium. J. Adv. Res..

[30] Patowary K., Patowary R., Kalita M.C., Deka S. (2017). Characterization of Biosurfactant Produced during Degradation of Hydrocarbons Using Crude Oil As Sole Source of Carbon. Front. Microbiol..

[31] Neethu C.S., Saravanakumar C., Purvaja R., Robin R.S., Ramesh R. (2019). Oil-Spill Triggered Shift in Indigenous Microbial Structure and Functional Dynamics in Different Marine Environmental Matrices. Sci. Rep..

[32] Ławniczak Ł., Woźniak-Karczewska M., Loibner A.P., Heipieper H.J., Chrzanowski Ł. (2020). Microbial Degradation of Hydrocarbons-Basic Principles for Bioremediation: A Review. Molecules.

[33] Ruberto L., Dias R., Lo Balbo A., Vazquez S.C., Hernandez E.A., Mac Cormack W.P. (2009). Influence of Nutrients Addition and Bioaugmentation on the Hydrocarbon Biodegradation of a Chronically Contaminated Antarctic Soil. J. Appl. Microbiol..

[34] Aranda E. (2016). Promising Approaches towards Biotransformation of Polycyclic Aromatic Hydrocarbons with Ascomycota Fungi. Curr. Opin. Biotechnol..

[35] Mawad A.M.M., Hesham A.E.-L., Khan S., Nawab J., Hesham A.E.-L., Upadhyay R.S., Sharma G.D., Manoharachary C., Gupta V.K. (2020). The Role of Fungi and Genes for the Removal of Environmental Contaminants from Water/Wastewater Treatment Plants. Fungal Biotechnology and Bioengineering.

[36] Zhou H., Huang X., Bu K., Wen F., Zhang D., Zhang C. (2019). Fungal Proliferation and Hydrocarbon Removal during Biostimulation of Oily Sludge with High Total Petroleum Hydrocarbon. Environ. Sci. Pollut. Res. Int..

[37] Grujić S., Vasić S., Radojević I., Čomić L., Ostojić A. (2017). Comparison of the Rhodotorula Mucilaginosa Biofilm and Planktonic Culture on Heavy Metal Susceptibility and Removal Potential. Water Air Soil Pollut..

[38] Nhi Cong L.T., Ngoc Mai C.T., Thanh V.T., Nga L.P., Minh N.N. (2014). Application of a Biofilm Formed by a Mixture of Yeasts Isolated in Vietnam to Degrade Aromatic Hydrocarbon Polluted Wastewater Collected from Petroleum Storage. Water Sci. Technol..

